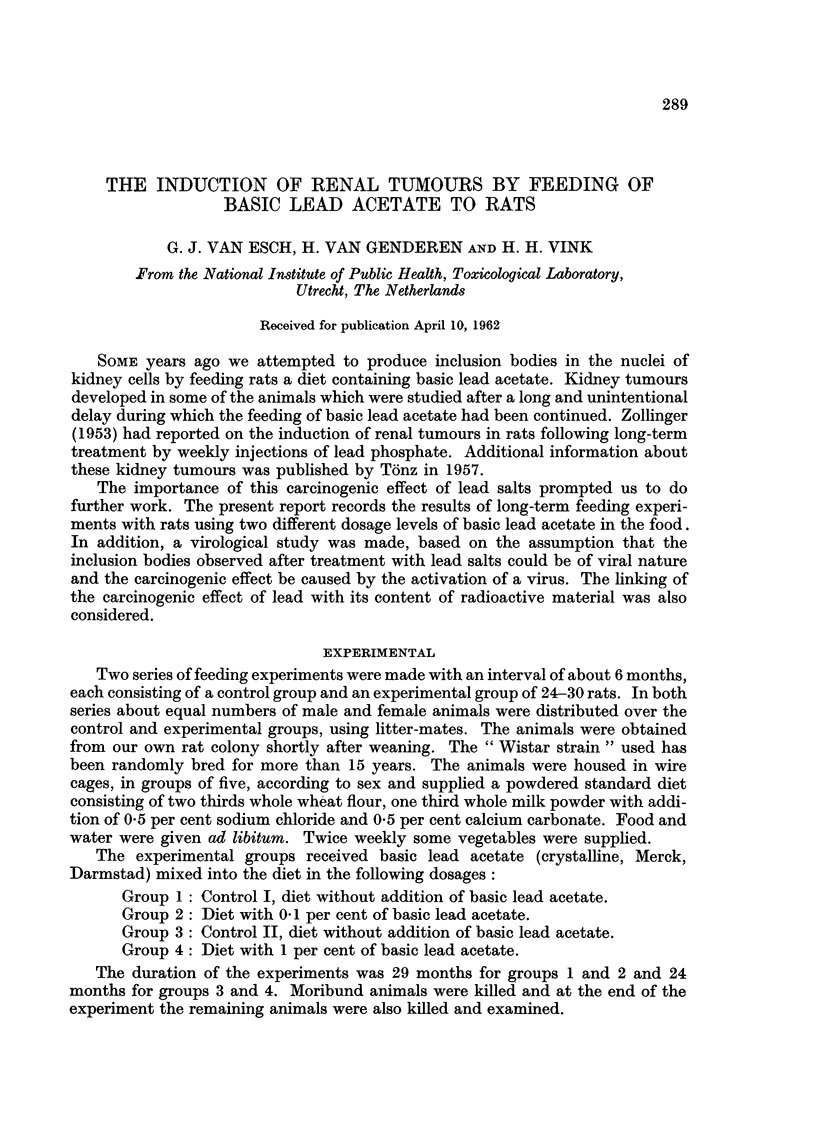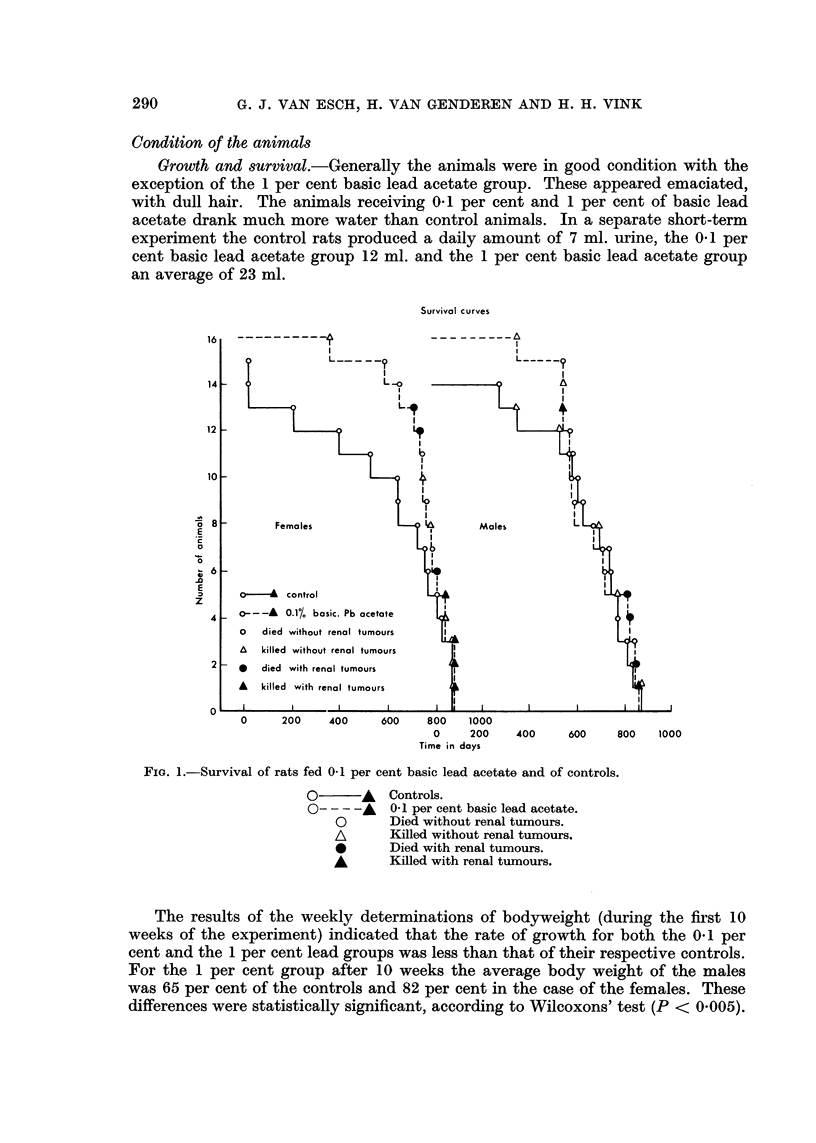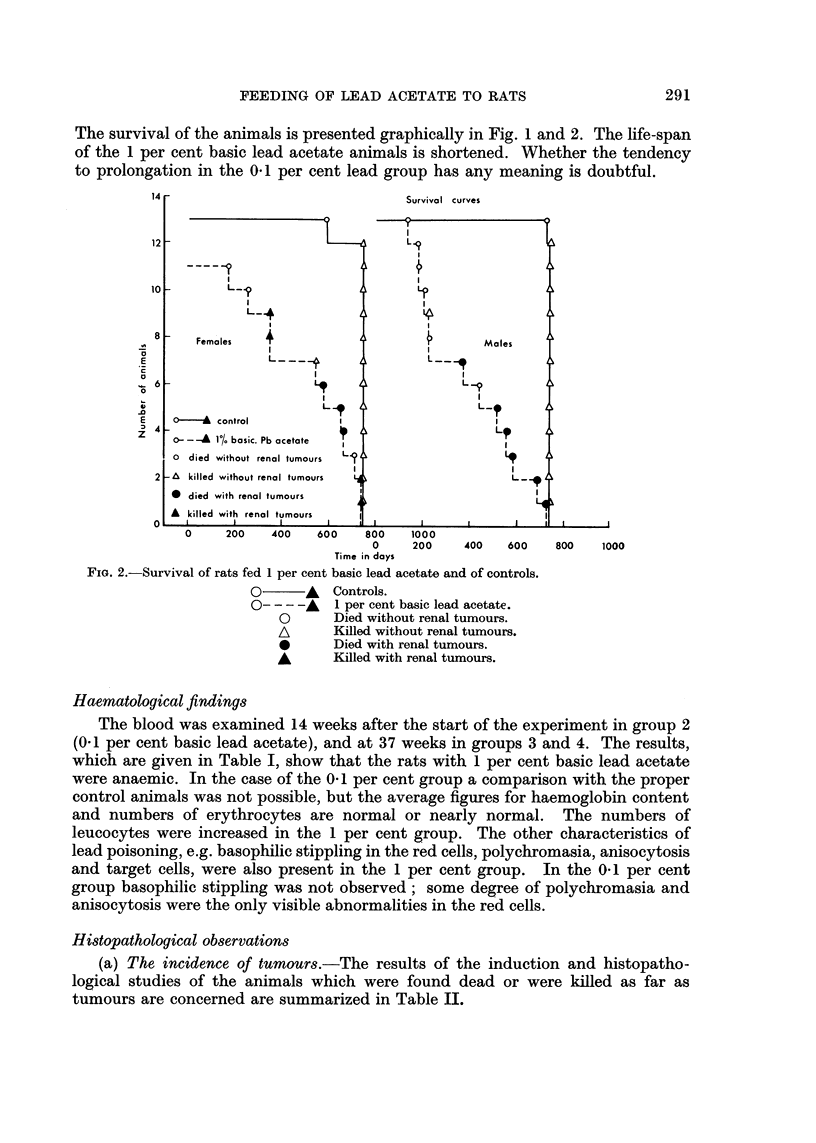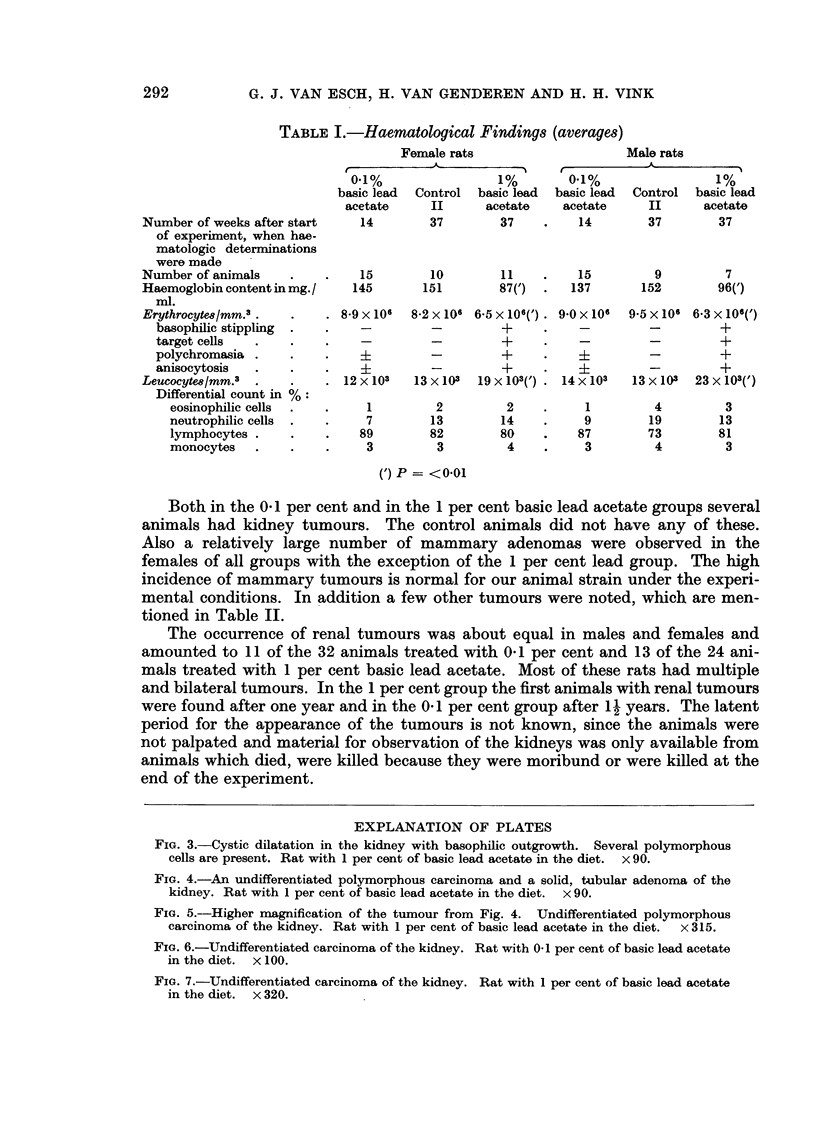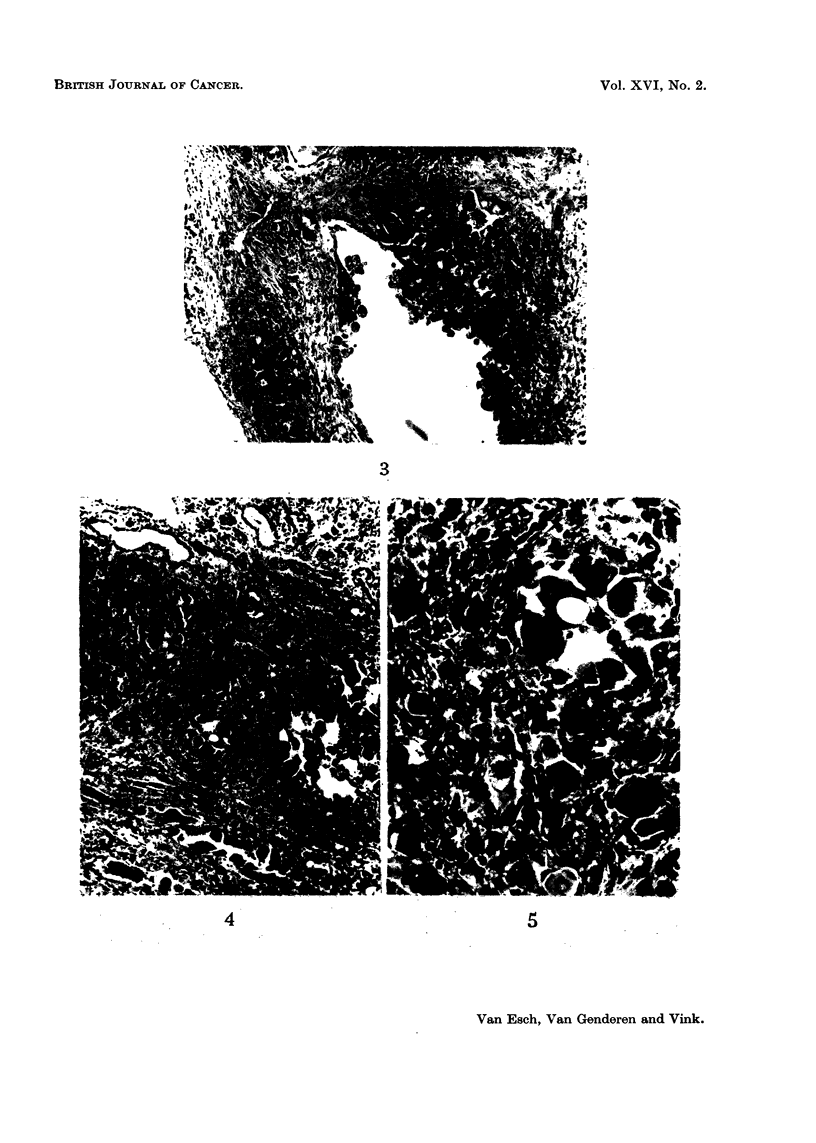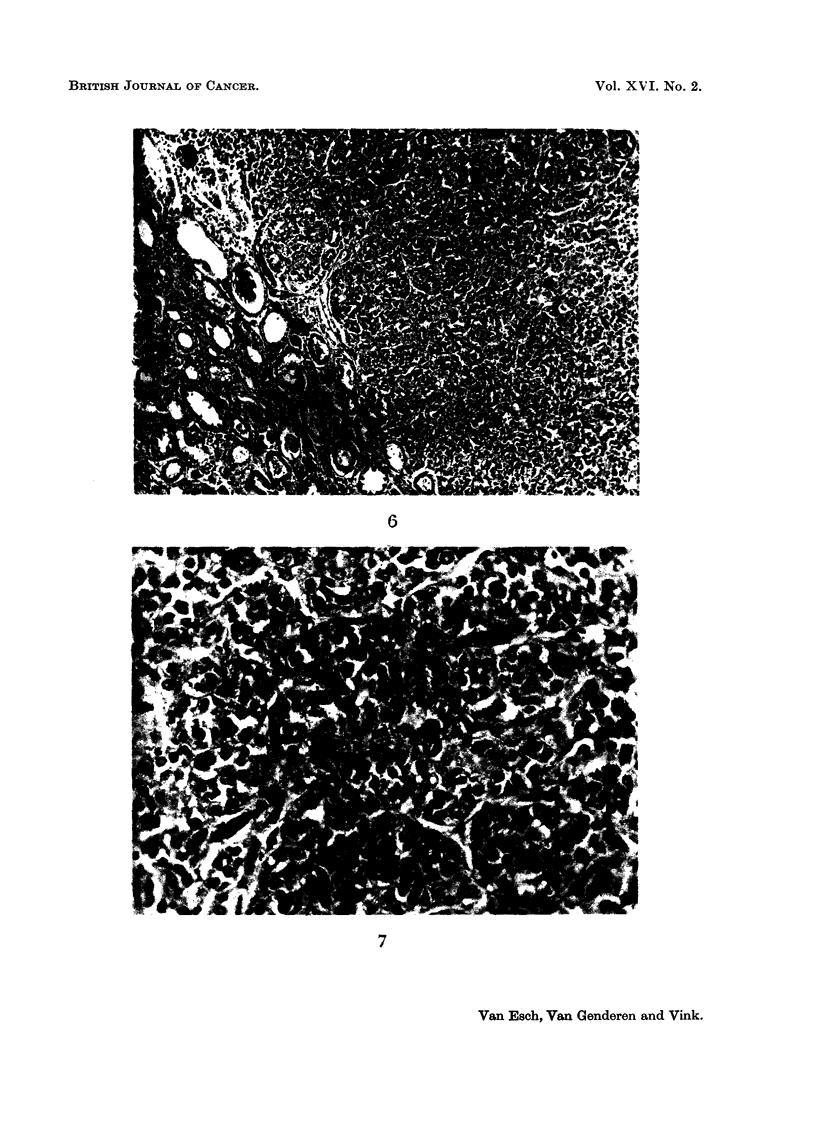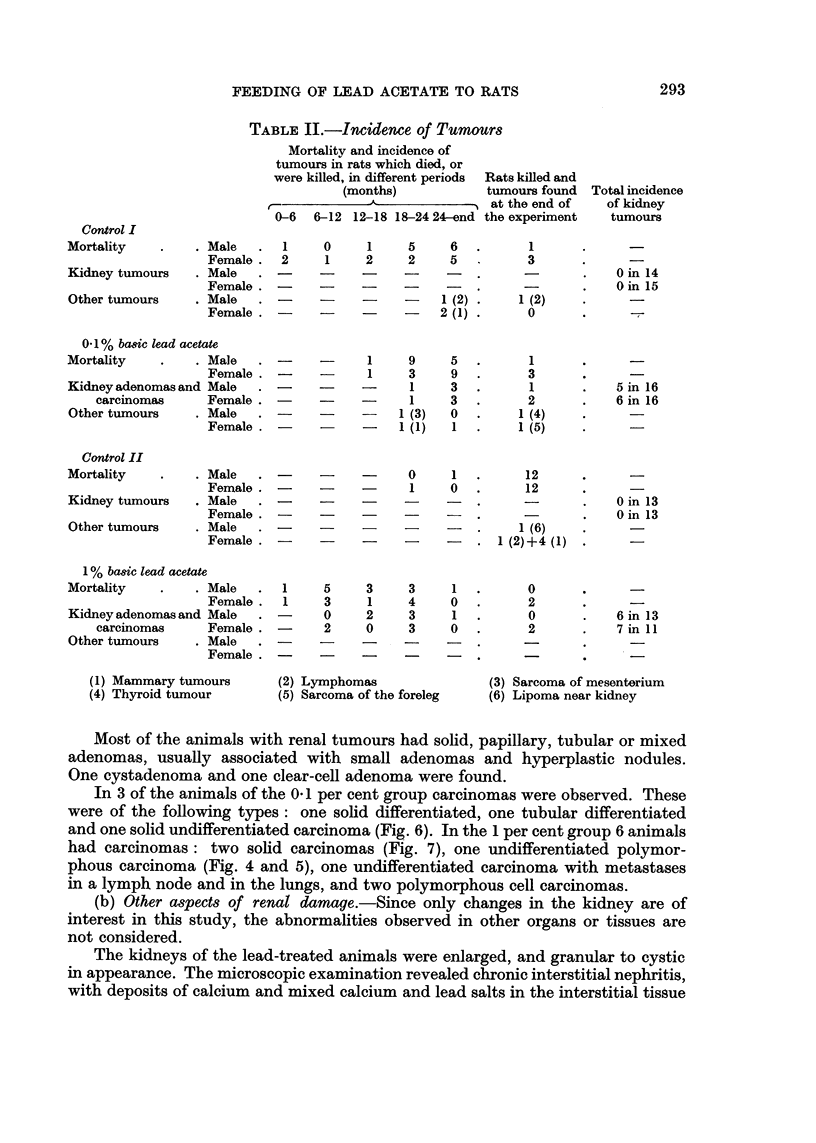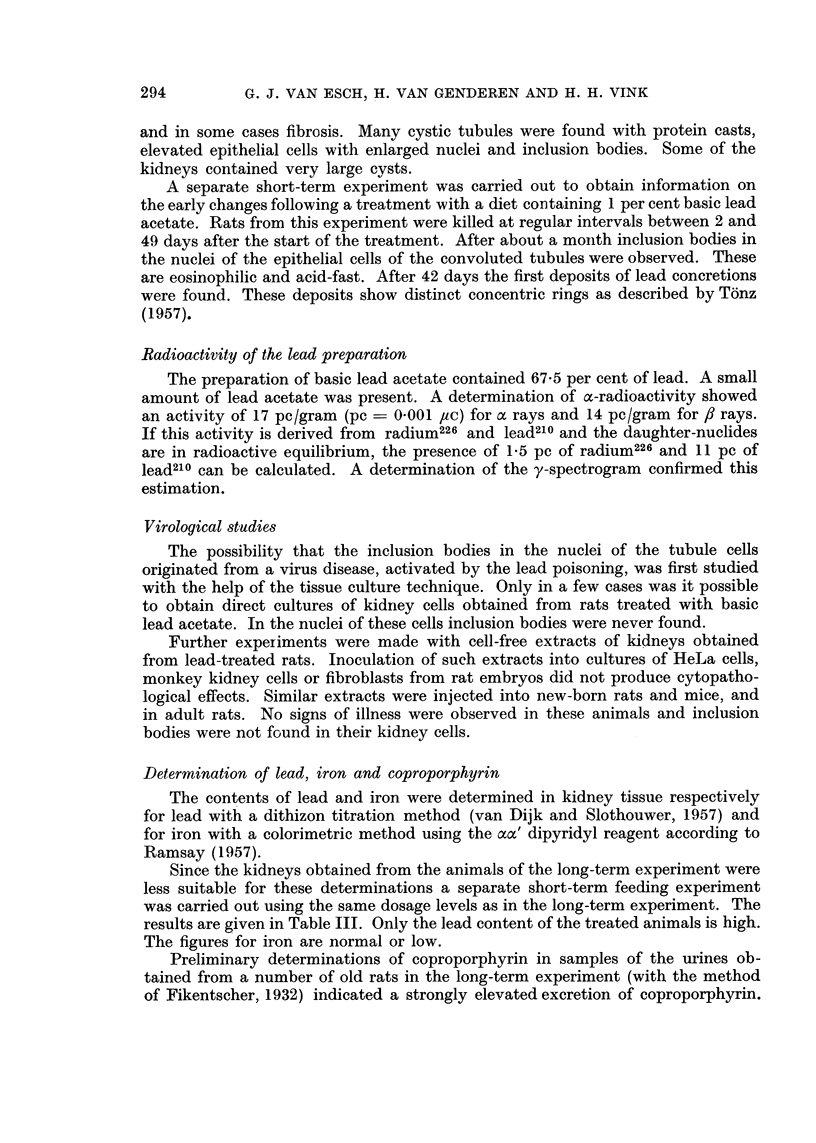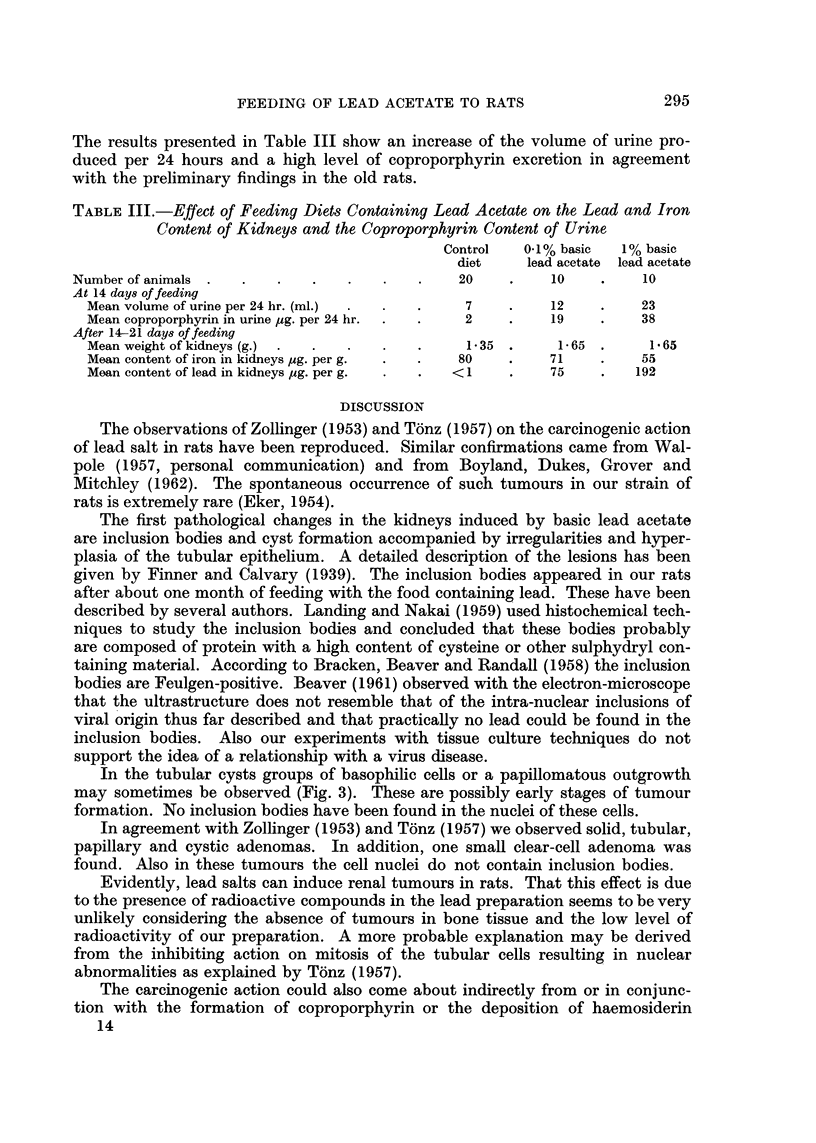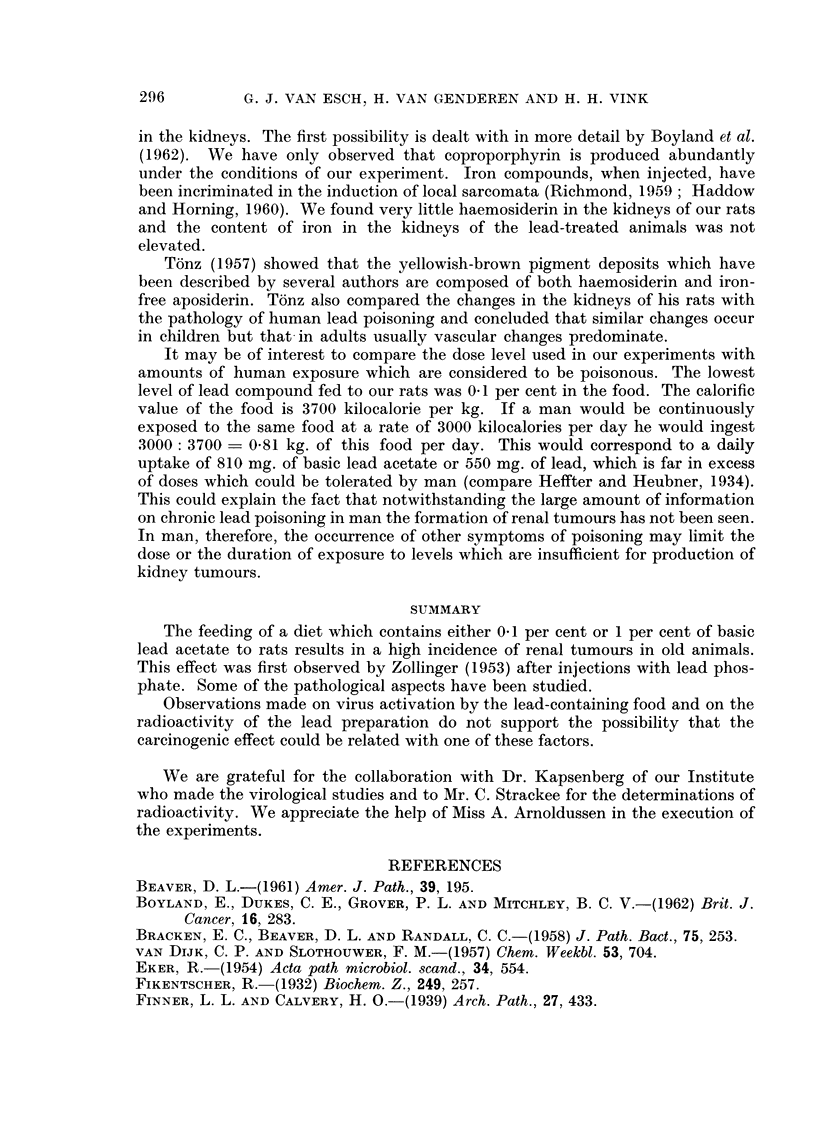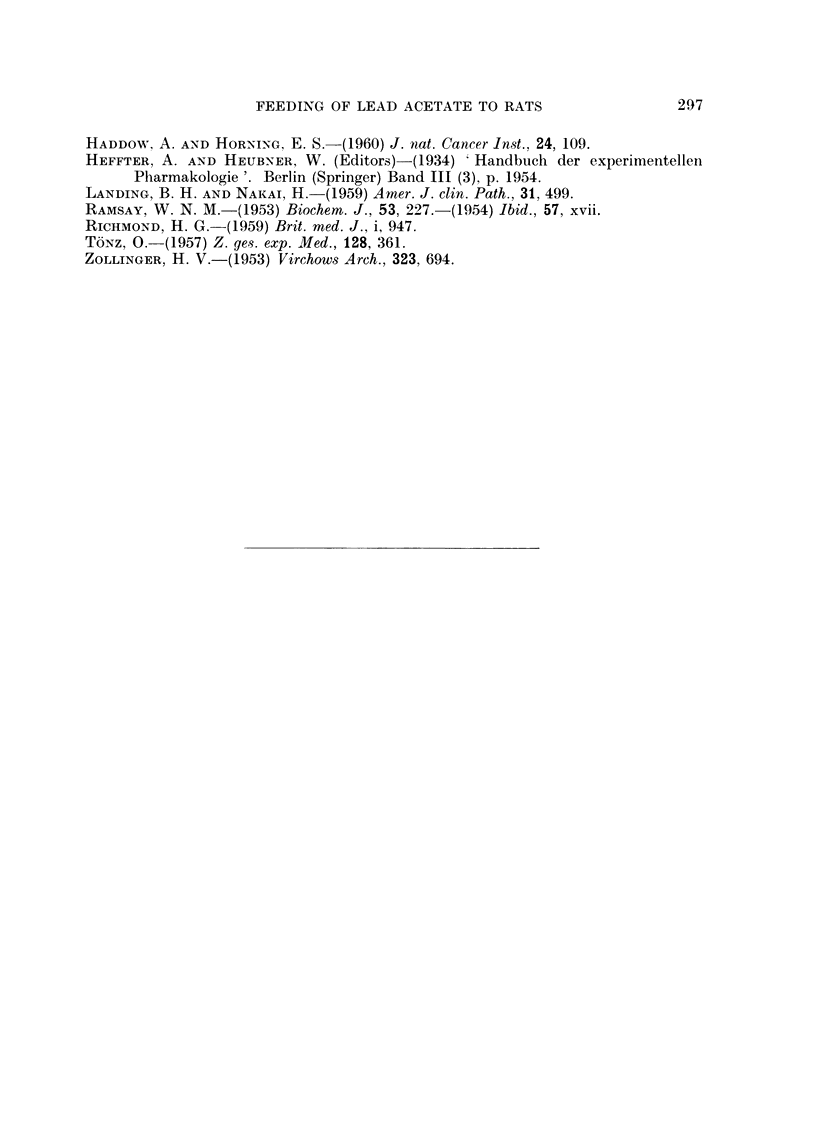# The Induction of Renal Tumours by Feeding of Basic Lead Acetate to Rats

**DOI:** 10.1038/bjc.1962.34

**Published:** 1962-06

**Authors:** G. J. van Esch, H. van Genderen, H. H. Vink

## Abstract

**Images:**


### THE INDUCTION OF RENAL TUMOURS BY FEEDING OF BASIC LEAD ACETATE TO RATS

G. J. VAN ESCH, H. VAN GENDEREN AND H. H. VINK

*From the National Institute of Public Health, Toxicological Laboratory, Utrecht, The Netherlands*

Received for publication April 10, 1962

SOME years ago we attempted to produce inclusion bodies in the nuclei of kidney cells by feeding rats a diet containing basic lead acetate. Kidney tumours developed in some of the animals which were studied after a long and unintentional delay during which the feeding of basic lead acetate had been continued. Zollinger (1953) had reported on the induction of renal tumours in rats following long-term treatment by weekly injections of lead phosphate. Additional information about these kidney tumours was published by Tönz in 1957.

The importance of this carcinogenic effect of lead salts prompted us to do further work. The present report records the results of long-term feeding experiments with rats using two different dosage levels of basic lead acetate in the food. In addition, a virological study was made, based on the assumption that the inclusion bodies observed after treatment with lead salts could be of viral nature and the carcinogenic effect be caused by the activation of a virus. The linking of the carcinogenic effect of lead with its content of radioactive material was also considered.

### EXPERIMENTAL

Two series of feeding experiments were made with an interval of about 6 months, each consisting of a control group and an experimental group of 24–30 rats. In both series about equal numbers of male and female animals were distributed over the control and experimental groups, using litter-mates. The animals were obtained from our own rat colony shortly after weaning. The " Wistar strain " used has been randomly bred for more than 15 years. The animals were housed in wire cages, in groups of five, according to sex and supplied a powdered standard diet consisting of two thirds whole wheat flour, one third whole milk powder with addition of 0·5 per cent sodium chloride and 0·5 per cent calcium carbonate. Food and water were given *ad libitum.* Twice weekly some vegetables were supplied.

The experimental groups received basic lead acetate (crystalline, Merck, Darmstad) mixed into the diet in the following dosages :

Group 1 : Control I, diet without addition of basic lead acetate.
Group 2 : Diet with 0·1 per cent of basic lead acetate.
Group 3 : Control II, diet without addition of basic lead acetate.
Group 4 : Diet with 1 per cent of basic lead acetate.

The duration of the experiments was 29 months for groups 1 and 2 and 24 months for groups 3 and 4. Moribund animals were killed and at the end of the experiment the remaining animals were also killed and examined.

*Condition of the animals*

*Growth and survival.*—Generally the animals were in good condition with the exception of the 1 per cent basic lead acetate group. These appeared emaciated, with dull hair. The animals receiving 0·1 per cent and 1 per cent of basic lead acetate drank much more water than control animals. In a separate short-term experiment the control rats produced a daily amount of 7 ml. urine, the 0·1 per cent basic lead acetate group 12 ml. and the 1 per cent basic lead acetate group an average of 23 ml.

FIG. 1.—Survival of rats fed 0·1 per cent basic lead acetate and of controls.

○——▲ Controls.
○- - - -▲ 0·1 per cent basic lead acetate.
○ Died without renal tumours.
△ Killed without renal tumours.
● Died with renal tumours.
▲ Killed with renal tumours.

The results of the weekly determinations of bodyweight (during the first 10 weeks of the experiment) indicated that the rate of growth for both the 0·1 per cent and the 1 per cent lead groups was less than that of their respective controls. For the 1 per cent group after 10 weeks the average body weight of the males was 65 per cent of the controls and 82 per cent in the case of the females. These differences were statistically significant, according to Wilcoxons' test ($P < 0{\cdot}005$).

The survival of the animals is presented graphically in Fig. 1 and 2. The life-span of the 1 per cent basic lead acetate animals is shortened. Whether the tendency to prolongation in the 0·1 per cent lead group has any meaning is doubtful.

Fig. 2.—Survival of rats fed 1 per cent basic lead acetate and of controls.

| | |
|---|---|
| ○———▲ | Controls. |
| ○– – – –▲ | 1 per cent basic lead acetate. |
| ○ | Died without renal tumours. |
| △ | Killed without renal tumours. |
| ● | Died with renal tumours. |
| ▲ | Killed with renal tumours. |

### *Haematological findings*

The blood was examined 14 weeks after the start of the experiment in group 2 (0·1 per cent basic lead acetate), and at 37 weeks in groups 3 and 4. The results, which are given in Table I, show that the rats with 1 per cent basic lead acetate were anaemic. In the case of the 0·1 per cent group a comparison with the proper control animals was not possible, but the average figures for haemoglobin content and numbers of erythrocytes are normal or nearly normal. The numbers of leucocytes were increased in the 1 per cent group. The other characteristics of lead poisoning, e.g. basophilic stippling in the red cells, polychromasia, anisocytosis and target cells, were also present in the 1 per cent group. In the 0·1 per cent group basophilic stippling was not observed ; some degree of polychromasia and anisocytosis were the only visible abnormalities in the red cells.

### *Histopathological observations*

(a) *The incidence of tumours.*—The results of the induction and histopathological studies of the animals which were found dead or were killed as far as tumours are concerned are summarized in Table II.

TABLE I.—*Haematological Findings (averages)*

| | Female rats | | | Male rats | | |
|---|---|---|---|---|---|---|
| | 0·1% basic lead acetate | Control II | 1% basic lead acetate | 0·1% basic lead acetate | Control II | 1% basic lead acetate |
| Number of weeks after start of experiment, when haematologic determinations were made | 14 | 37 | 37 | 14 | 37 | 37 |
| Number of animals | 15 | 10 | 11 | 15 | 9 | 7 |
| Haemoglobin content in mg./ml. | 145 | 151 | 87(′) | 137 | 152 | 96(′) |
| *Erythrocytes/mm.*$^3$ | $8{\cdot}9\times10^6$ | $8{\cdot}2\times10^6$ | $6{\cdot}5\times10^6$(′) | $9{\cdot}0\times10^6$ | $9{\cdot}5\times10^6$ | $6{\cdot}3\times10^6$(′) |
| basophilic stippling | − | − | + | − | − | + |
| target cells | − | − | + | − | − | + |
| polychromasia | ± | − | + | ± | − | + |
| anisocytosis | ± | − | + | ± | − | + |
| *Leucocytes/mm.*$^3$ | $12\times10^3$ | $13\times10^3$ | $19\times10^3$(′) | $14\times10^3$ | $13\times10^3$ | $23\times10^3$(′) |
| Differential count in %: | | | | | | |
| eosinophilic cells | 1 | 2 | 2 | 1 | 4 | 3 |
| neutrophilic cells | 7 | 13 | 14 | 9 | 19 | 13 |
| lymphocytes | 89 | 82 | 80 | 87 | 73 | 81 |
| monocytes | 3 | 3 | 4 | 3 | 4 | 3 |

(′) $P = <0{\cdot}01$

Both in the 0·1 per cent and in the 1 per cent basic lead acetate groups several animals had kidney tumours. The control animals did not have any of these. Also a relatively large number of mammary adenomas were observed in the females of all groups with the exception of the 1 per cent lead group. The high incidence of mammary tumours is normal for our animal strain under the experimental conditions. In addition a few other tumours were noted, which are mentioned in Table II.

The occurrence of renal tumours was about equal in males and females and amounted to 11 of the 32 animals treated with 0·1 per cent and 13 of the 24 animals treated with 1 per cent basic lead acetate. Most of these rats had multiple and bilateral tumours. In the 1 per cent group the first animals with renal tumours were found after one year and in the 0·1 per cent group after 1½ years. The latent period for the appearance of the tumours is not known, since the animals were not palpated and material for observation of the kidneys was only available from animals which died, were killed because they were moribund or were killed at the end of the experiment.

---

EXPLANATION OF PLATES

FIG. 3.—Cystic dilatation in the kidney with basophilic outgrowth. Several polymorphous cells are present. Rat with 1 per cent of basic lead acetate in the diet. ×90.

FIG. 4.—An undifferentiated polymorphous carcinoma and a solid, tubular adenoma of the kidney. Rat with 1 per cent of basic lead acetate in the diet. ×90.

FIG. 5.—Higher magnification of the tumour from Fig. 4. Undifferentiated polymorphous carcinoma of the kidney. Rat with 1 per cent of basic lead acetate in the diet. ×315.

FIG. 6.—Undifferentiated carcinoma of the kidney. Rat with 0·1 per cent of basic lead acetate in the diet. ×100.

FIG. 7.—Undifferentiated carcinoma of the kidney. Rat with 1 per cent of basic lead acetate in the diet. ×320.

3

4 5

6

7

TABLE II.—*Incidence of Tumours*

| | | Mortality and incidence of tumours in rats which died, or were killed, in different periods (months) | | | | | Rats killed and tumours found at the end of the experiment | Total incidence of kidney tumours |
|---|---|---|---|---|---|---|---|---|
| | | 0–6 | 6–12 | 12–18 | 18–24 | 24–end | | |
| *Control I* | | | | | | | | |
| Mortality | Male | 1 | 0 | 1 | 5 | 6 | 1 | — |
| | Female | 2 | 1 | 2 | 2 | 5 | 3 | — |
| Kidney tumours | Male | — | — | — | — | — | — | 0 in 14 |
| | Female | — | — | — | — | — | — | 0 in 15 |
| Other tumours | Male | — | — | — | — | 1 (2) | 1 (2) | — |
| | Female | — | — | — | — | 2 (1) | 0 | — |
| *0·1% basic lead acetate* | | | | | | | | |
| Mortality | Male | — | — | 1 | 9 | 5 | 1 | — |
| | Female | — | — | 1 | 3 | 9 | 3 | — |
| Kidney adenomas and carcinomas | Male | — | — | — | 1 | 3 | 1 | 5 in 16 |
| | Female | — | — | — | 1 | 3 | 2 | 6 in 16 |
| Other tumours | Male | — | — | — | 1 (3) | 0 | 1 (4) | — |
| | Female | — | — | — | 1 (1) | 1 | 1 (5) | — |
| *Control II* | | | | | | | | |
| Mortality | Male | — | — | — | 0 | 1 | 12 | — |
| | Female | — | — | — | 1 | 0 | 12 | — |
| Kidney tumours | Male | — | — | — | — | — | — | 0 in 13 |
| | Female | — | — | — | — | — | — | 0 in 13 |
| Other tumours | Male | — | — | — | — | — | 1 (6) | — |
| | Female | — | — | — | — | — | 1 (2)+4 (1) | — |
| *1% basic lead acetate* | | | | | | | | |
| Mortality | Male | 1 | 5 | 3 | 3 | 1 | 0 | — |
| | Female | 1 | 3 | 1 | 4 | 0 | 2 | — |
| Kidney adenomas and carcinomas | Male | — | 0 | 2 | 3 | 1 | 0 | 6 in 13 |
| | Female | — | 2 | 0 | 3 | 0 | 2 | 7 in 11 |
| Other tumours | Male | — | — | — | — | — | — | — |
| | Female | — | — | — | — | — | — | — |

(1) Mammary tumours (2) Lymphomas (3) Sarcoma of mesenterium
(4) Thyroid tumour (5) Sarcoma of the foreleg (6) Lipoma near kidney

Most of the animals with renal tumours had solid, papillary, tubular or mixed adenomas, usually associated with small adenomas and hyperplastic nodules. One cystadenoma and one clear-cell adenoma were found.

In 3 of the animals of the 0·1 per cent group carcinomas were observed. These were of the following types : one solid differentiated, one tubular differentiated and one solid undifferentiated carcinoma (Fig. 6). In the 1 per cent group 6 animals had carcinomas : two solid carcinomas (Fig. 7), one undifferentiated polymorphous carcinoma (Fig. 4 and 5), one undifferentiated carcinoma with metastases in a lymph node and in the lungs, and two polymorphous cell carcinomas.

(b) *Other aspects of renal damage.*—Since only changes in the kidney are of interest in this study, the abnormalities observed in other organs or tissues are not considered.

The kidneys of the lead-treated animals were enlarged, and granular to cystic in appearance. The microscopic examination revealed chronic interstitial nephritis, with deposits of calcium and mixed calcium and lead salts in the interstitial tissue and in some cases fibrosis. Many cystic tubules were found with protein casts, elevated epithelial cells with enlarged nuclei and inclusion bodies. Some of the kidneys contained very large cysts.

A separate short-term experiment was carried out to obtain information on the early changes following a treatment with a diet containing 1 per cent basic lead acetate. Rats from this experiment were killed at regular intervals between 2 and 49 days after the start of the treatment. After about a month inclusion bodies in the nuclei of the epithelial cells of the convoluted tubules were observed. These are eosinophilic and acid-fast. After 42 days the first deposits of lead concretions were found. These deposits show distinct concentric rings as described by Tönz (1957).

*Radioactivity of the lead preparation*

The preparation of basic lead acetate contained 67·5 per cent of lead. A small amount of lead acetate was present. A determination of $\alpha$-radioactivity showed an activity of 17 pc/gram (pc = 0·001 $\mu$c) for $\alpha$ rays and 14 pc/gram for $\beta$ rays. If this activity is derived from radium$^{226}$ and lead$^{210}$ and the daughter-nuclides are in radioactive equilibrium, the presence of 1·5 pc of radium$^{226}$ and 11 pc of lead$^{210}$ can be calculated. A determination of the $\gamma$-spectrogram confirmed this estimation.

*Virological studies*

The possibility that the inclusion bodies in the nuclei of the tubule cells originated from a virus disease, activated by the lead poisoning, was first studied with the help of the tissue culture technique. Only in a few cases was it possible to obtain direct cultures of kidney cells obtained from rats treated with basic lead acetate. In the nuclei of these cells inclusion bodies were never found.

Further experiments were made with cell-free extracts of kidneys obtained from lead-treated rats. Inoculation of such extracts into cultures of HeLa cells, monkey kidney cells or fibroblasts from rat embryos did not produce cytopathological effects. Similar extracts were injected into new-born rats and mice, and in adult rats. No signs of illness were observed in these animals and inclusion bodies were not found in their kidney cells.

*Determination of lead, iron and coproporphyrin*

The contents of lead and iron were determined in kidney tissue respectively for lead with a dithizon titration method (van Dijk and Slothouwer, 1957) and for iron with a colorimetric method using the $\alpha\alpha'$ dipyridyl reagent according to Ramsay (1957).

Since the kidneys obtained from the animals of the long-term experiment were less suitable for these determinations a separate short-term feeding experiment was carried out using the same dosage levels as in the long-term experiment. The results are given in Table III. Only the lead content of the treated animals is high. The figures for iron are normal or low.

Preliminary determinations of coproporphyrin in samples of the urines obtained from a number of old rats in the long-term experiment (with the method of Fikentscher, 1932) indicated a strongly elevated excretion of coproporphyrin.

The results presented in Table III show an increase of the volume of urine produced per 24 hours and a high level of coproporphyrin excretion in agreement with the preliminary findings in the old rats.

TABLE III.—*Effect of Feeding Diets Containing Lead Acetate on the Lead and Iron Content of Kidneys and the Coproporphyrin Content of Urine*

| | Control diet | 0·1% basic lead acetate | 1% basic lead acetate |
|---|---|---|---|
| Number of animals | 20 | 10 | 10 |
| *At* 14 *days of feeding* | | | |
| Mean volume of urine per 24 hr. (ml.) | 7 | 12 | 23 |
| Mean coproporphyrin in urine μg. per 24 hr. | 2 | 19 | 38 |
| *After* 14–21 *days of feeding* | | | |
| Mean weight of kidneys (g.) | 1·35 | 1·65 | 1·65 |
| Mean content of iron in kidneys μg. per g. | 80 | 71 | 55 |
| Mean content of lead in kidneys μg. per g. | $<1$ | 75 | 192 |

### DISCUSSION

The observations of Zollinger (1953) and Tönz (1957) on the carcinogenic action of lead salt in rats have been reproduced. Similar confirmations came from Walpole (1957, personal communication) and from Boyland, Dukes, Grover and Mitchley (1962). The spontaneous occurrence of such tumours in our strain of rats is extremely rare (Eker, 1954).

The first pathological changes in the kidneys induced by basic lead acetate are inclusion bodies and cyst formation accompanied by irregularities and hyperplasia of the tubular epithelium. A detailed description of the lesions has been given by Finner and Calvary (1939). The inclusion bodies appeared in our rats after about one month of feeding with the food containing lead. These have been described by several authors. Landing and Nakai (1959) used histochemical techniques to study the inclusion bodies and concluded that these bodies probably are composed of protein with a high content of cysteine or other sulphydryl containing material. According to Bracken, Beaver and Randall (1958) the inclusion bodies are Feulgen-positive. Beaver (1961) observed with the electron-microscope that the ultrastructure does not resemble that of the intra-nuclear inclusions of viral origin thus far described and that practically no lead could be found in the inclusion bodies. Also our experiments with tissue culture techniques do not support the idea of a relationship with a virus disease.

In the tubular cysts groups of basophilic cells or a papillomatous outgrowth may sometimes be observed (Fig. 3). These are possibly early stages of tumour formation. No inclusion bodies have been found in the nuclei of these cells.

In agreement with Zollinger (1953) and Tönz (1957) we observed solid, tubular, papillary and cystic adenomas. In addition, one small clear-cell adenoma was found. Also in these tumours the cell nuclei do not contain inclusion bodies.

Evidently, lead salts can induce renal tumours in rats. That this effect is due to the presence of radioactive compounds in the lead preparation seems to be very unlikely considering the absence of tumours in bone tissue and the low level of radioactivity of our preparation. A more probable explanation may be derived from the inhibiting action on mitosis of the tubular cells resulting in nuclear abnormalities as explained by Tönz (1957).

The carcinogenic action could also come about indirectly from or in conjunction with the formation of coproporphyrin or the deposition of haemosiderin in the kidneys. The first possibility is dealt with in more detail by Boyland *et al.* (1962). We have only observed that coproporphyrin is produced abundantly under the conditions of our experiment. Iron compounds, when injected, have been incriminated in the induction of local sarcomata (Richmond, 1959 ; Haddow and Horning, 1960). We found very little haemosiderin in the kidneys of our rats and the content of iron in the kidneys of the lead-treated animals was not elevated.

Tönz (1957) showed that the yellowish-brown pigment deposits which have been described by several authors are composed of both haemosiderin and iron-free aposiderin. Tönz also compared the changes in the kidneys of his rats with the pathology of human lead poisoning and concluded that similar changes occur in children but that in adults usually vascular changes predominate.

It may be of interest to compare the dose level used in our experiments with amounts of human exposure which are considered to be poisonous. The lowest level of lead compound fed to our rats was 0·1 per cent in the food. The calorific value of the food is 3700 kilocalorie per kg. If a man would be continuously exposed to the same food at a rate of 3000 kilocalories per day he would ingest 3000 : 3700 = 0·81 kg. of this food per day. This would correspond to a daily uptake of 810 mg. of basic lead acetate or 550 mg. of lead, which is far in excess of doses which could be tolerated by man (compare Heffter and Heubner, 1934). This could explain the fact that notwithstanding the large amount of information on chronic lead poisoning in man the formation of renal tumours has not been seen. In man, therefore, the occurrence of other symptoms of poisoning may limit the dose or the duration of exposure to levels which are insufficient for production of kidney tumours.

### SUMMARY

The feeding of a diet which contains either 0·1 per cent or 1 per cent of basic lead acetate to rats results in a high incidence of renal tumours in old animals. This effect was first observed by Zollinger (1953) after injections with lead phosphate. Some of the pathological aspects have been studied.

Observations made on virus activation by the lead-containing food and on the radioactivity of the lead preparation do not support the possibility that the carcinogenic effect could be related with one of these factors.

We are grateful for the collaboration with Dr. Kapsenberg of our Institute who made the virological studies and to Mr. C. Strackee for the determinations of radioactivity. We appreciate the help of Miss A. Arnoldussen in the execution of the experiments.

### REFERENCES

BEAVER, D. L.—(1961) *Amer. J. Path.*, **39**, 195.

BOYLAND, E., DUKES, C. E., GROVER, P. L. AND MITCHLEY, B. C. V.—(1962) *Brit. J. Cancer*, **16**, 283.

BRACKEN, E. C., BEAVER, D. L. AND RANDALL, C. C.—(1958) *J. Path. Bact.*, **75**, 253.

VAN DIJK, C. P. AND SLOTHOUWER, F. M.—(1957) *Chem. Weekbl.* **53**, 704.

EKER, R.—(1954) *Acta path microbiol. scand.*, **34**, 554.

FIKENTSCHER, R.—(1932) *Biochem. Z.*, **249**, 257.

FINNER, L. L. AND CALVERY, H. O.—(1939) *Arch. Path.*, **27**, 433.

Haddow, A. and Horning, E. S.—(1960) *J. nat. Cancer Inst.*, **24**, 109.
Heffter, A. and Heubner, W. (Editors)—(1934) 'Handbuch der experimentellen Pharmakologie'. Berlin (Springer) Band III (3), p. 1954.
Landing, B. H. and Nakai, H.—(1959) *Amer. J. clin. Path.*, **31**, 499.
Ramsay, W. N. M.—(1953) *Biochem. J.*, **53**, 227.—(1954) *Ibid.*, **57**, xvii.
Richmond, H. G.—(1959) *Brit. med. J.*, i, 947.
Tönz, O.—(1957) *Z. ges. exp. Med.*, **128**, 361.
Zollinger, H. V.—(1953) *Virchows Arch.*, **323**, 694.